# Incidence of Sepsis-Induced Coagulopathy (INSIC) Trial: Study Protocol of a Combined Retrospective and Prospective, Multicenter, International, Cross-Sectional, Longitudinal, and Epidemiological Observational Trial

**DOI:** 10.3390/jcm14124222

**Published:** 2025-06-13

**Authors:** Thomas Schmoch, Patrick Möhnle, Markus A. Weigand, Maximilian Dietrich, Jonas Gregorius, Sandra Frank, Josef Briegel, David I. Radke, Michael Bauer, Frank Bloos, Patrick Meybohm, Holger Bogatsch, Thorsten Brenner

**Affiliations:** 1 Department of Anesthesiology and Intensive Care Medicine, University Hospital Essen, University Duisburg-Essen, 45141 Essen, Germany; jonas.gregorius@uk-essen.de (J.G.); thorsten.brenner@uk-essen.de (T.B.); 2 Department of Anesthesiology and Intensive Care Medicine, Hôpitaux Robert Schuman–Hôpital Kirchberg, L-2540 Luxembourg City, Luxembourg; 3 Division of Transfusion Medicine, Cell Therapeutics and Haemostaseology, LMU University Hospital, LMU Munich, 81377 Munich, Germany; patrick.moehnle@med.uni-muenchen.de; 4 Department of Anesthesiology, LMU University Hospital, LMU Munich, 81377 Munich, Germany; sandra.frank@med.uni-muenchen.de (S.F.); josef.briegel@med.uni-muenchen.de (J.B.); 5 Department of Anesthesiology, Medical Faculty Heidelberg, Heidelberg University, Im Neuenheimer Feld 420, 69120 Heidelberg, Germany; markus.weigand@med.uni-heidelberg.de (M.A.W.); maximilian.dietrich@med.uni-heidelberg.de (M.D.); 6 Department of Anesthesiology and Intensive Care Medicine, University Medical Center Schleswig-Holstein, Campus Kiel, 24105 Kiel, Germany; david.radke@uksh.de; 7 Center for Sepsis Control and Care, Jena University Hospital, 07747 Jena, Germany; michael.bauer@med.uni-jena.de; 8 Department of Anesthesiology and Intensive Care Medicine, Jena University Hospital, 07747 Jena, Germany; frank.bloos@med.uni-jena.de; 9 Department of Anaesthesiology, Intensive Care, Emergency and Pain Medicine, University Hospital Würzburg, 97080 Würzburg, Germany; meybohm_p@ukw.de; 10 Institute for Medical Informatics, Statistics and Epidemiology (IMISE) and Clinical Trial Centre Leipzig, 04107 Leipzig, Germany

**Keywords:** sepsis, septic shock, sepsis-induced coagulopathy, incidence, prevalence

## Abstract

**Background/Objectives:** Sepsis and septic shock are the most severe forms of infection. Due to the intensive cross-talk of the coagulation and immune system, coagulopathies regularly occur in sepsis. The International Society on Thrombosis and Hemostasis refers to these coagulation abnormalities as sepsis-induced coagulopathies (SICs). The presence of SICs can be assessed using the SIC score. In parallel, a score for “sepsis-associated coagulopathy” (SAC) was introduced that, in contrast to the SIC score, allows coagulopathy to be classified according to its severity. In the past, multicenter, randomized controlled trials have repeatedly failed to prove the efficacy of specific therapeutic measures targeting SICs or SACs. This could potentially be explained by insufficient knowledge about the prevalence and incidence of SIC and the rate of spontaneous recovery. The Incidence of Sepsis-Induced Coagulopathy (INSIC) trial is intended to address this problem. **Methods:** The aim of the INSIC trial is to measure the incidence and prevalence of SIC—in addition to the rate of spontaneous SIC recoveries—during the first days of sepsis treatment to provide a solid base of data for future interventional trials. We aim to include all patients with sepsis treated in any one of 150 participating intensive care units in 100 hospitals located in Austria, Luxembourg, and Germany to determine the prevalence of SIC (on day 1 of the study period as well as on three selected days in the fourth quarter of each year from 2019 to 2024). SIC incidence will be assessed over a 14-day period starting on 10 March 2025. Secondary endpoints are 28-day survival and the occurrence of severe thromboembolic events and bleeding. In addition, each of the aforementioned outcome parameters will be assessed for correlation with the severity classification of the SAC score. **Conclusions:** The INSIC trial is the first study to determine the prevalence as well as the incidence of SIC, to prospectively examine the course of SIC longitudinally over a 14-day period, and to determine the rate of spontaneous recoveries within 72 h under standard treatment in a large cohort of patients. This information will provide a sound basis for future studies.

## 1. Introduction

Whenever wounds occur in nature, the human body relies on the simultaneous and rapid prevention of both further blood loss and the invasion of germs into the bloodstream. As a result, there is so much cross-talk between coagulation mediators and the innate immune response to infection that experts consider the two systems to be one [1]. The role of monocytes, neutrophil granulocytes, and the complement system are just three examples of this complicated cross-linking: activated monocytes express tissue factor (TF), factor III of the coagulation system. When neutrophil granulocytes are activated as part of the innate immune response, they eject, among other things, so-called neutrophil extracellular traps (NETs), in which bacteria become trapped and can then be cleared by phagocytes. These NETs activate factor XII of the coagulation cascade. Activated platelets in turn also activate the complement system and vice versa [1]. This seems evolutionarily useful as long as these interactions remain localized around wound areas. In the case of sepsis and septic shock, this intense cross-talk may become a problem. Defined as “life-threatening organ dysfunction caused by a dysregulated host response to infection”, sepsis and septic shock are the most severe forms of infection [2]. Coagulation disorders (coagulopathies) are an integral part of sepsis [3]. To address this phenomenon clinically, the International Society on Thrombosis and Hemostasis (ISTH) provided a screening tool for recognizing sepsis-induced coagulopathy (SIC) in 2017 named the SIC score [4] (Table 1). The aim of this tool is to detect SIC at an early stage before it develops into the severe form of overt disseminated intravascular coagulation (DIC) [4,5]. The latter occurs when a systematic, uncontrolled activation of coagulation leads to an excessive consumption of coagulation factors, resulting in deficiency and subsequent diffuse bleeding. Simultaneously, multiple microthrombi and thromboembolisms may occur. In order to describe different degrees of severity of coagulopathies in sepsis, almost simultaneously with the SIC score, the “sepsis-associated coagulopathy” (SAC) score (Table 1) was introduced, distinguishing “mild”, “moderate”, and “severe” SAC [6]. From a clinical perspective, it appears crucial to identify SIC at an early stage, as this is the only way to potentially intervene therapeutically and prevent obvious DIC with diffuse bleeding and thrombosis.

In a secondary analysis of two multicenter studies published in 2023, we were able to show that at least 20–25% of patients with sepsis or septic shock were affected by SIC, and that SIC was associated with increased morbidity as well as mortality [7,8,9]. However, the exact incidence of SIC is difficult to estimate, and the prevalence of SIC in retrospective studies varies from 20 to 80%, depending on the severity of the disease and the pre-selection of the respective study cohort [4,9,10,11]. This high level of variance makes a reliable calculation of expected case numbers for randomized controlled interventional trials (RCTs) extremely difficult [4,9,10,11]. This is most likely one of the main reasons why a number of RCTs that intended to modulate blood coagulation in sepsis therapeutically and thus to reduce sepsis mortality failed [12,13,14]. Most recently, an RCT investigating the effects of soluble recombinant thrombomodulin in patients with sepsis and SAC failed in 2019 because approximately 20% of the patients who had coagulopathy at the time of screening had experienced a spontaneous remission of SAC under standard sepsis therapy by the time the study drug was administered for the first time [14]. Nevertheless, coagulopathies in septic patients are strongly associated with increased morbidity and mortality [4,9,10], and addressing coagulopathies therapeutically may be a critical factor in reducing sepsis mortality.

The aim of this multicenter observational study is to measure the incidence of SIC and the rate of spontaneous SIC recovery in the first days of sepsis treatment, and thus to provide a solid foundation of data to inform further interventional RCTs.

### 1.1. Objectives

The primary outcome of the INSIC trial is the SIC incidence rate within a 14-day observation period (new cases/1000 ICU days). Moreover, we aim to determine the point prevalence of SIC on day 1 of the study period and on three reference days in the fourth quarter of each year from 2019 to 2023. The secondary outcome parameters are as follows: (I) length of stay in intensive care unit (ICU) until transfer to a normal ward; (II) ICU mortality rate (in all patients and in subgroups of patients with sepsis, septic shock, and SIC according to the different scores); (III) 28-day hospital mortality rate (in all patients and in subgroups of patients with sepsis, septic shock, and SIC according to the different scores); (IV) SIC recovery rate 72 h after SIC onset; (V) thromboembolic events after sepsis onset (in sepsis, septic shock, or SIC) up to day 28, death, or hospital discharge (whichever occurs first); and (VI) relevant bleeding without surgical trauma (non-trauma or surgery-related major bleeding causing a drop in hemoglobin of <1 g/dL) after the onset of sepsis (in sepsis, septic shock, or SIC) until day 28, death, or hospital discharge (whichever occurs first). (VII) Each of the above-mentioned outcome parameters will also be tested for correlation with the different degrees of severity of SAC (according to the SAC score) (Table 1), which allows for a more differentiated assessment of the severity of coagulopathy.

### 1.2. Trial Design

The INSIC trial is a combined retrospective and prospective, multicenter, international, cross-sectional, longitudinal and epidemiological observational study. This study protocol follows the standard protocol items in accordance with the Recommendations for Observational Trials (STROBE) guidelines.

## 2. Methods

### 2.1. Study Setting

The INSIC trial is a combined retrospective and prospective, multicenter, international, cross-sectional, longitudinal and epidemiological observational study. The aim of the study is to have 150 ICUs across 100 hospitals in Austria, Luxembourg, and Germany participate. The INSIC trial will be conducted by the network of the SepNet Critical Care Trials Group of the German Sepsis Foundation.

### 2.2. Eligibility Criteria

General patient data is collected from all adult patients treated in one of the participating intensive care units during the observation period (see Section 2.5). Patients are screened for sepsis on a daily basis. Patients are included in the detailed data collection if they fulfill all of the following criteria:

1. The patient must be treated in a participating ICU.
2. The patient must be ≥18 years of age.
3. The patient must be diagnosed with sepsis or septic shock [2]:
  - Definition of sepsis: Acute suspected or confirmed infection plus a sepsis-induced increase in SOFA score by ≥2 points compared with baseline (last-documented value in the ICU or approximate baseline value in cases with known pre-existing conditions, e.g., documented SOFA impairment due to renal insufficiency, COPD, thrombocytopenia, or pre-existing brain damage). In cases where no pre-existing conditions are identified, the pre-existing SOFA score can be assumed to be “0”.
  - Definition of septic shock: Sepsis plus persisting hypotension requiring vasopressors to maintain MAP ≥ 65 mm Hg plus lactate ≥ 2 mmol/L after an initial bolus volume of 30 mL/kg body weight (bw).

### 2.3. Interventions

There are no interventions.

### 2.4. Outcomes

The primary outcome parameters of the INSIC trial are as follows:

(1) The incidence rate of SIC within a 14-day observation period (new cases/1000 patient days).
(2) The point prevalence of SIC on day 1 of the study period and on three reference days in the 4th quarter of each year from 2019 to 2023.

The secondary outcome parameters of the INSIC trial are as follows:

(1) Length of stay in the ICU until transfer to a normal ward.
(2) ICU mortality rate (in all patients and in the subgroups of patients with sepsis, septic shock, SIC, or SAC (mild, moderate, severe) according to the different scores).
(3) Mortality rate until day 28, hospital death, or hospital discharge (whatever occurs first, in all patients and in the subgroups of patients with sepsis, septic shock, SIC, or SAC (mild, moderate, severe) according to the different scores).
(4) SIC and SAC (mild, moderate, severe) recovery rate 72 h after SIC or SAC onset. The time point was chosen as the 72 h period reflects a critical window during which early death in sepsis or a clinical response to sepsis therapy can be expected [15].
(5) Thromboembolic events after sepsis onset (in sepsis, septic shock, SIC, or SAC (mild, moderate, severe)) up to day 28, death, or hospital discharge (whichever occurs first).
(6) Relevant bleeding without surgical trauma (hemoglobin loss >1 g/dL within 24 h, due to bleeding rather than dilution) after sepsis onset (in sepsis, septic shock, SIC, or SAC (mild, moderate, severe)) until day 28, death, or hospital discharge (whichever occurs first).

### 2.5. Procedure and Data Collection

**Figure 1:**
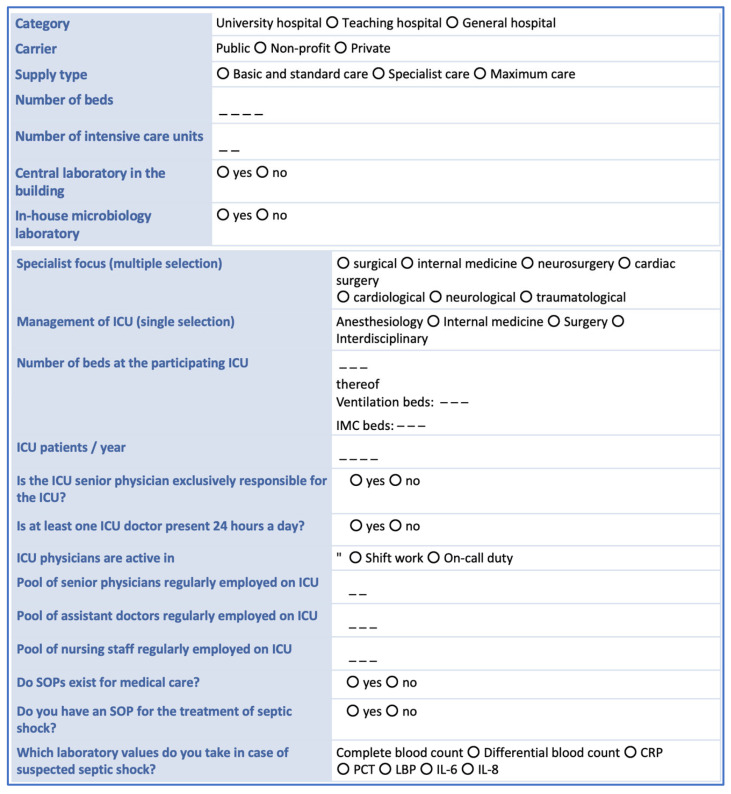
Hospital structural data collected from each study center. CRP = C-reactive protein; ICU = intensive care unit, LBP = lipopolysaccharide-binding protein, IL = interleukin; PCT = procalcitonin, SOP = standard operating procedure.

We will collect general structural data (see Figure 1) for each participating study center. All patients treated in the participating ICUs in a 14-day period will be included. Notably, only data generated in the context of standard patient care will be collected. For each patient treated in the participating ICUs, the following data will be collected: gender, age, date and time of hospital admission, referring ward or hospital (if applicable), Simplified Acute Physiology Score (SAPS) II, date and time of hospital discharge, patient status at discharge (alive, dead, returning home, or going to a care facility). In addition, for each patient we intend to collect data describing the dates and times of ICU admission and discharge as well as the type of admission (surgery—planned or emergency; medical treatment—planned or emergency) and the survival status at ICU discharge.

**Figure 2:**
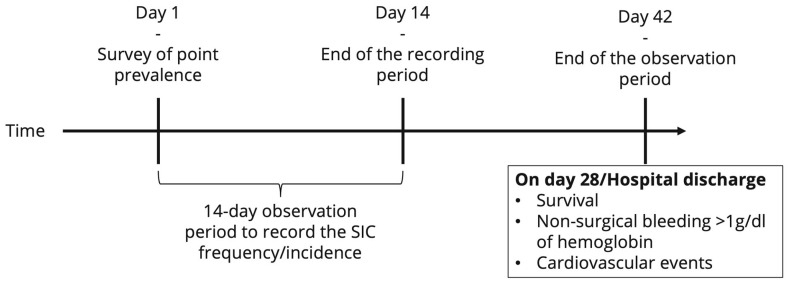
Overview of the timeline of the INSIC trial. SIC = sepsis-induced coagulopathy.

For each patient diagnosed with sepsis or septic shock during the observation period, more detailed information will be collected (date and time of sepsis onset, date and time of septic shock onset, infectious focus, causative pathogen(s), and secondary infections (super- and/or nosocomial infections and/or infections of a further organ system)). Furthermore, investigators will be asked to extract data from the hospital information system describing additional patient characteristics. These include the American Society of Anesthesiologists (ASA) classification [16], the Revised Cardiac Risk Index (RCRI) [17] score, the status of and, if applicable, the reason for pre-existing anticoagulation therapy or antiplatelet therapy, coagulopathy, and severe liver disease. Furthermore, information on past episodes of heparin-induced thrombocytopenia (HIT) type II will be collected. For all patients hospitalized with sepsis in the ICU on the first day of the observation period, it will be determined whether SIC was present before the start of the observation period. Furthermore, for each patient diagnosed with sepsis or septic shock during the observation period, on the day of sepsis diagnosis (day 0), on days 1–4, and on the day of sepsis discharge, the following measures will be collected: the sequential sepsis-related organ failure assessment (SOFA) score [2], the international normalized ratio (INR), and platelet count. For further exploratory analyses, the following parameters are recorded where available: anti-factor Xa activity, activated partial thromboplastin time (aPTT), prothrombin time, D-dimer level, fibrinogen concentration, thrombin–antithrombin complex (TAT) concentration, thrombin fragment F1.2 concentration, and measures from thrombelastometric testing. Likewise for exploratory analyses, the following parameters are collected: the levels of inflammation markers (C-reactive protein (CRP), procalcitonin (PCT), and interleukin-(IL) 6), as well as the dose of anticoagulant administered, the amount of fluid infused (per day), the amount of blood products transfused (per day), and the administered amount of coagulation factors and desmopressin (per day), determined at the same time points. Using the collected data and the SIC scores, all patients with sepsis will be tested for SIC at least once a day. Additionally, SIC scores (exact scores) will be recorded daily until day 14 after sepsis onset or discharge from the ICU (whatever occurs first). The date and time of the first SIC occurrence will be registered. The data collected will also be used to calculate the severity of SAC, according to Lyons et al. [6]. Finally, until day 14 after the onset of sepsis, each patient will be checked for thromboembolic events and non-trauma or surgery-related major bleeding (causing a drop in hemoglobin of <1 g/dL) once a day. In addition, on the 28th day after sepsis onset, it will be determined whether cardiovascular events, thromboembolic events, or non-trauma or surgery-related major bleeding (causing a drop in hemoglobin of <1 g/dL) occurred during the second 14 days of follow-up monitoring (yes/no recording for the second 14 days). Figure 2 provides an overview of the planned timeline. The time frames were chosen on the basis of balancing efforts and benefits. Since it is currently assumed that SIC occurs in the early phase of sepsis due to a dysregulation of the interaction of the innate immune response with the coagulation system, a special focus was placed on the first days, i.e., day 0 (sepsis onset) and the following 4 days. The aim is to identify possible early warning signs of SIC. Sepsis is usually treated anti-infectively for 7–14 days. The first occurrence of SIC after day 14 was considered very unlikely. The experts involved in the study planning were of the opinion that the occurrence of SIC after day 14 was most likely due to a secondary infection (which triggers a new sepsis episode) or another sepsis-independent reason. It was decided to use the 28-day mortality rate, as it is frequently collected in sepsis studies and thus allows an approximate comparison of the mortality of this cohort with other study cohorts. In the aforementioned effort to balance efforts and benefits, it was decided to investigate the potential effects of SIC on thromboembolism and bleeding risk up to day 28.

### 2.6. Evaluation of the Change in SIC Prevalence

For three selected days, October 15, November 15, and December 15 of the years 2019–2023, at all ICUs of the participating hospitals, two types of data will be collected from the hospital information systems: (1) The total number of patients who were in the ICU on that day (00:00 to 23:59) will be documented (regardless of whether this day was during a continuous stay, at admission, or at discharge/death; the total number of patients can therefore be higher than the number of beds). (2) The total number of patients with sepsis or septic shock on that day (00:00 to 23:59) will be collected, and both SIC and SAC scores will be calculated for each patient with sepsis. The 15th of each month was chosen arbitrarily. The 4th quarter was chosen because the 4th quarter of 2019 was before the start of the coronavirus disease 2019 (COVID-19) pandemic and the 4th quarters of 2020 and 2021 (in Germany) were clearly in a pandemic wave.

### 2.7. Sample Size

It is not possible to calculate a required sample size, as to undertake this the prevalence and incidence of SIC in Austria, Luxembourg, and Germany would need to be known and this study is the first to survey these measures. The “incidence of severe sepsis and septic shock in German intensive care units” INSEP study of the SepNet Critical Care Trials Group of the German Sepsis Society served as the basis for the expected estimates of prevalence and incidence [18]. In the INSEP study, the incidence of severe sepsis and the incidence of septic shock were recorded over a period of 4 weeks in 133 intensive care units at 95 hospitals in Germany. Secondarily, the point prevalences of severe sepsis and septic shock were recorded. The study was carried out according to the definition of Sepsis-2 that was valid at the time of the study. The intensive care units participated on a voluntary basis and are not a representative sample of Germany. Almost 12,000 patients were screened, of whom around 1500 had severe sepsis or septic shock. There are currently no data on the incidence of SIC. A Japanese study found that probably 30–60% of patients with sepsis and septic shock develop SIC (depending on which scoring tool is used to make the diagnosis) [19].

The INSIC trial is very similar to the INSEP trial [18]. In contrast to the INSEP trial, the focus of the INSIC trial presented here is on determining the prevalence and incidence of SIC. The study was planned as part of the SepNet Critical Care Trials Group in close consultation with the potential study centers. No case fees can be paid out, meaning that the INSIC trial is only possible thanks to the commitment of the study centers. In order to balance the workload and benefits as well as possible, the observation period was limited to 14 days. Based on the distribution of the ICUs participating in the INSEP study, the following prognosis results are predicted for the INSIC study: Within the two-week study period of the INSIC study, around 6700 patients can be expected to be treated in the participating ICUs. Based on the figures from the INSEP study, it is concluded that around 850 [95% confidence interval, approx. 800–900] patients with sepsis and septic shock can be expected at the hospitals over 14 days. Based on the data from Japan [19], around 250–500 patients with SIC are likely to be identified. Regarding the collection of data on SIC prevalence from the years 2019–2023, the identification of approximately 2100–2200 patients per survey date can be assumed. Depending on the composition of the participating ICUs (level of care, medical specialization), possible seasonal disease clusters, and the distribution of disease severity in the participating ICUs, the SIC incidence may potentially be over- or underestimated. This could limit the validity of the data and its generalizability. These effects are attempted to be reduced by the size of the study. The possible significance of these effects can be estimated as soon as the data are available.

### 2.8. Data Collection Methods

Data collection will be carried out by the physicians/investigators of the participating ICUs. Once a day, the information required for the study is extracted from the data collected as part of standard patient care and entered into the electronic case report form (eCRF/REDCap^®^, Vanderbilt University, Nashville, TN, USA [20,21]). The investigators of the participating ICUs are responsible for ensuring that all parts of the eCRFs are filled in correctly. The completed eCRF must be approved by the investigator or by a designated sub-investigator.

### 2.9. Data Management

The Institute for Medical Informatics, Statistics and Epidemiology (IMISE, Leipzig, Germany) and the Clinical Trial Centre Leipzig (Leipzig, Germany) are responsible for creating and validating the eCRF. The data will be managed and analyzed according to the appropriate standard operating procedures (SOPs) that are valid in the IMISE/ZKS. According to §13 of the Good Clinical Practice Ordinance [22], all important trial documents (e.g., Case Report Forms (CRFs)) will be archived for at least 10 years after the completion of the clinical trial.

### 2.10. Statistical Methods

All patients who do not have SIC at the beginning of their individual observation period will be included in the calculation of the cumulative observation period for determining the incidence rate of SIC. The observation period for determining the incidence rate of SIC ends at the time of diagnosis of SIC or discharge from the ICU (depending on which event occurs first), but at the latest at the end of the 14-day observation period. The incidence rates for sepsis and septic shock are determined in the same way. To determine the incidence density, the number of incident patients is divided by the cumulative sum of the individual observation days of all patients included in the incidence calculation. The incidence density is thus calculated according to the following formula:

$$\frac{\mathrm{Number}\;\mathrm{of}\;\mathrm{incidental}\;\mathrm{cases}}{\begin{array}{l} \mathrm{Sum}\;\mathrm{of}\;\mathrm{the}\;\mathrm{individual}\;\mathrm{observation}\;\mathrm{period}\;\mathrm{across}\;\mathrm{all}\;\mathrm{cases} \end{array}}$$

The corresponding 95% confidence interval is calculated based on a Poisson distribution. The incidence rates between the individual strata will be compared in pairs.

The point prevalences for SIC, sepsis, and septic shock will be determined on the first day of the observation period by calculating the ratio of the number of affected patients to the total number of patients in the ICUs on that day. The Wilson score method will be used to determine the associated 95% confidence interval. Using χ^2^ tests (or Fisher’s exact tests), the point prevalences of the individual strata will be compared. The prevalences of SIC, sepsis, and septic shock in the retrospective data collection will be determined analogously.

The available data will be described by specifying the median [interquartile range (IQR)] or the absolute (relative) frequency. Depending on the type of characteristic being analyzed, the different groups will be compared using the χ^2^ test (or Fisher’s exact test, if necessary), the Mann–Whitney U test, or the *t*-test. The Kaplan–Meier method will be used to determine survival times, and the log-rank test will be used for the relevant group comparisons.

A logistic regression model will be used to investigate mortality and describe it using odds ratios (including the associated 95% confidence intervals). Only variables that prove to be significant in the univariate analysis will be transferred to a multivariate model.

Multivariate logistic regression will be performed to compare the survival of SIC-positive and SIC-negative patients while adjusting for age, sex, the use of anticoagulants, and the SIC-adapted SOFA score at sepsis onset (i.e., the time of sepsis diagnosis). To calculate the SIC score, the SOFA score will be adapted according to Iba et al. [4] (see also Table 1). Two-sided *p* values will be used.

### 2.11. Data Monitoring

To enable standardized data collection across all centers, the investigators are trained via video conferencing before the start of the study. In addition, written material and a frequently asked questions (FAQ) list are available for all investigators. Furthermore, regular video conferences are offered during the ongoing study to answer questions about the study process and data collection. The FAQ list will be updated regularly. Moreover, the RedCap database used automatically checks data plausibility (according to tables of plausible values stored in advance) and completeness. Automated queries and notes are issued to the user. However, as only data collected as part of standard patient care may be entered, queries due to missing data may be ignored by the investigators. Moreover, central monitoring is carried out by the staff of IMISE Leipzig. In the event of anomalies that indicate, e.g., systematic errors in data entry, the relevant study center is contacted. As this is a trial that is purely observational in nature, no professional monitoring is planned. All local investigators are invited to keep in close contact with the principal investigator (email, telephone, video call apps) to ensure that the trial is conducted according to the protocol and regulatory requirements. Based on the experience of the INSEP study, which used a very similar study protocol and was conducted to a large extent at the same study centers by the same people, a high compliance rate regarding data collection across participating study centers is expected [18].

### 2.12. Harms

Due to the purely non-interventional nature of the INSIC trial, study-related adverse events (AEs) are not to be expected.

### 2.13. Auditing

No scheduled audits by the sponsor are intended to be conducted. In cases where the relevant authorities need to conduct an on-site inspection or audit, the investigator must ensure the availability of all documents and support this work at any time.

### 2.14. Ethics

The study will be conducted according to SOPs designed to ensure that all involved parties abide by the principles of Good Clinical Practice (GCP) [22,23] and the Declaration of Helsinki [24]. Moreover, the INSIC trial will be carried out in accordance with local statutory and implementing provisions.

### 2.15. Approval of the Institutional Review Board

Patients will not be enrolled at each site until there has been approval from the relevant institutional review board (IRB). The first IRB to approve the study completion was the Ethics Committee of the Medical Faculty of the University Duisburg-Essen (Votum no. 21-9969-BO).

### 2.16. Protocol Amendments

Changes to the protocol must be made in writing and require the approval of all signatories of the protocol. Subsequent amendments also require a positive assessment from the relevant IRB.

### 2.17. Consent or Assent

The requirement for written informed consent was waived by the IRB, as in the INSIC trial only data collected during standard patient care are included. Moreover, all data processing will be anonymous, and prevalence and incidence calculations are only possible if all patients are included.

### 2.18. Confidentiality

All collected data will be handled in accordance with the provisions of the General Data Protection Regulation (GDPR) of the European Union and the European Council [25]. All data containing the identity of the patient will only be accessible to the attending doctors and nursing staff; for all other investigators involved in the study, this data is encrypted and will use a pseudonym. Only the investigators at the respective study site are able to assign a pseudonym to a patient’s name, and only for as long as needed to complete data collection. After the 28-day follow-up, the data sets will be completely anonymized so that decryption is no longer possible. All data that will be processed and used in subsequent publications will be strictly anonymous.

## 3. Discussion

SIC is significantly associated with increased morbidity and mortality [9]. Studies from Japan and China and also a subgroup analysis of the SCARLET (Sepsis Coagulopathy Asahi Recombinant LE Thrombomodulin) trial suggest a causal influence of coagulopathy on mortality [4,14,26]. Several studies on the possible effects of anticoagulation, using various compounds, have been undertaken in this area. In a retrospective study by Yamakawa et al., anticoagulation was clearly associated with reduced mortality, at least in patients with sepsis and overt DIC and in patients with a SOFA score of 13–18 [27]. A subgroup analysis of the SCARLET trial showed that the study drug thrombomodulin probably has a more pronounced positive effect when used only in patients with high thrombin fragment 1.2 levels [28]. In the SCARLET trial, 800 patients with severe sepsis (according to the sepsis-2 definition) and SAC were randomized; half of the patients received recombinant soluble thrombomodulin and the other half received a placebo [14]. The study failed to show a difference in its primary endpoint of 28-day mortality [14]. However, a subgroup analysis showed that in approximately 20% of the included patients, SAC had already resolved between the patients’ time of study inclusion and the time of the first administration of the medication [14]. When considering only the remaining 80% of patients who actually met the inclusion criteria when the study drug was first administered, the difference in survival is significant; however, the study was not powered for this setting. In this way, the SCARLET trial highlights problems that have probably contributed to the failure of a whole series of RCTs: [13,29,30,31]. We know too little about the incidence of sepsis-induced coagulopathies and too little about their spontaneous course and the factors that determine whether spontaneous recovery occurs or SIC contributes to morbidity and mortality. The INSIC trial is designed to provide new insights into the actual incidence and course of SIC. This may lay the foundation for more precise planning of interventional RCTs aimed at modulating coagulation in sepsis. The strength of our work lies in the large number of participating centers. Since so far only retrospective data on SIC prevalence in partly pre-selected groups are available [4,9,10,11,32], INSIC is intended to be the first to measure SIC incidence across a broad range of different patients with sepsis. In addition, the INSIC trial should provide insights into the proportion of patients who experience spontaneous recovery within the first 72 h of standard sepsis treatment (according to the guidelines of the Surviving Sepsis Campaign) [33]. However, we will also have to face some limitations. Firstly, it is expected that more university hospitals than small hospitals will participate. This could potentially lead to a bias towards more severe cases, but also towards more aggressive treatment. For example, difficult cases with a potentially good prognosis could be referred more often from smaller hospitals to university hospitals, where maximal therapy is provided, whereas smaller hospitals would more often continue to treat patients for whom, due to age for example, not all maximal therapeutic measures are desirable or indicated. However, this bias could also be an advantage in using the results of the INSIC trial to plan interventional studies, as these are traditionally conducted at university hospitals due to their need for human resources. Secondly, since we only request data collected as part of standard care, we are limited to the blood values that are typically measured. Rarely performed tests, such as thrombin fragment 1.2 or thrombin–antithrombin complex levels, will rarely be reported, meaning we will likely obtain only small sample sizes for these data. Overall, the INSIC trial is the first study to determine the incidence of SIC and to prospectively examine the course of SIC longitudinally over a 14-day period in a large sample size. This provides a sound basis for planning future RCTs.

### 3.1. Justification for Enrolment of Participants Not Capable of Giving Consent

For the purely observational INSIC trial, patient information leaflets and informed consent forms will not be issued for the following reasons: (1) Data collection is based on data from routine clinical practice; additional parameters are not collected. The data will be analyzed anonymously and without the possibility of drawing conclusions about individual patients, and results will be presented in a scientific publication. No further study-associated measures/activities will be carried out. (2) The INSIC trial is a prevalence and incidence study in which a large number of patients must be included who are not capable of giving consent at the required time of inclusion due to the underlying disease (sepsis or septic shock). Acute treatment will be carried out in these cases as part of emergency care in accordance with the patient’s presumed will, and setting up urgent guardianship for consent to collect anonymized data from clinical practice is not deemed to be justified. (3) The potential benefits in carrying out the research project on anonymized data that are routinely collected in clinical care outweigh the interests of subjects in being excluded from data processing. The data to be collected as part of the study described here will also be suitable for identifying potential target groups for drug therapies for SIC and thus improving medical care in Austria, Luxembourg, and Germany, and possibly worldwide. As this is purely an observational study, patients do not need to fear any impairment of their care as a result of participating in the study.

#### 3.1.1. Trial Status

Protocol version: Version: 1.2 (15 January 2025)

Patient and data recruitment started in March 2025 in all study centers that have obtained approval from their IRB.

#### 3.1.2. Trial Registration

DRKS-ID: DRKS00035249 (registered 9 October 2024)

https://drks.de/search/en/trial/DRKS00035249.

## Figures and Tables

**Table 1:** Sepsis-induced and sepsis-associated coagulopathy scores.

SIC Score Iba et al. [4]			SAC Score Lyons et al. [6]
		Points	
Platelet count SIC subscore (PSSC) (platelet count (PLC) [1/nL]	>150/nL	0	**Mild SAC** 1.2 ≤ INR < 1.4 **and** 100 > PLC ≤ 150
	100 to <150/nL	1	
	<100/nL	2	
International Normalized Ratio (INR) SIC subscore (ISSC) [ ]		0	
	≥1.2 to <1.4	1	
	≥1.4	2	
SOFA subscore (truncated SOFA score, including the respiratory, cardiocirculatory, hepatic, and renal subscore) [Points]	0	0	
	1	1	
	≥2	2	**Moderate SAC:** 1.4 ≤ INR < 1.6 **or** 80 ≥ PLC ≤ 100;
No SIC		<4 points	**Severe SAC** INR ≥ 1.6 **or** PLC < 80
SIC		≥4 points (with PT-INR plus platelet count exceeding 2)	

ISSC = international normalized ratio (INR) SIC subscore; PLC = platelet count; PSSC = platelet sepsis-induced coagulopathy subscore; SAC = sepsis-associated coagulopathy; SIC = sepsis-induced coagulopathy; SOFA = sequential (sepsis-related) organ failure assessment.

## Data Availability

Access to study data may be granted following a formal request to the Study Management Committee (Studienleitkommission) of the SepNet Critical Care Trials Group for approval. It can be contacted at sepsis@med.uni-jena.de.

## List of Abbreviations

AE	adverse event
aPTT	activated partial thromboplastin time
BDSG	Federal Data Protection Act
CNER	Comité National d’Ethique de Recherche
COPD	chronic obstructive pulmonary disease
CRP	C-reactive protein
DRKS	Deutsches Register Klinikscher Studien (German Clinical Trials Register)
DVT	deep vein thrombosis
eCRF	electronic case report form
F1.2	thrombin fragment 1.2
Hb	hemoglobin
ICU	intensive care unit
IL	interleukin
INR	international normalized ratio
INSIC	incidence of sepsis-induced coagulopathy
INSEP	incidence of severe sepsis and septic shock
IMISE	Institute for Medical Informatics, Statistics and Epidemiology
IU	international units
LAE	lung artery embolism
LBP	lipopolysaccharide-binding protein
MAP	mean arterial pressure
PCT	procalcitonin
PI	primary investigator
RBC	red blood cells
RCT	randomized controlled trial
SAE	serious adverse event
SAC	sepsis-associated coagulopathy
SIC	sepsis-induced coagulopathy
SOFA	sepsis-related organ failure assessment
TAT	thrombin–antithrombin complex
TC	thrombocyte concentrate
